# The genome sequence of the European peacock butterfly, *Aglais io* (Linnaeus, 1758)

**DOI:** 10.12688/wellcomeopenres.17204.1

**Published:** 2021-10-12

**Authors:** Konrad Lohse, Alexander Mackintosh, Roger Vila, Darwin Tree of Life Barcoding collective, Wellcome Sanger Institute Tree of Life programme, Wellcome Sanger Institute Scientific Operations: DNA Pipelines collective, Tree of Life Core Informatics collective, Darwin Tree of Life Consortium

**Affiliations:** 1 Institute of Evolutionary Biology, University of Edinburgh, Edinburgh, UK; 2 Institut de Biologia Evolutiva (CSIC - Universitat Pompeu Fabra), Barcelona, Spain

**Keywords:** Aglais io, European peacock butterfly, genome sequence, chromosomal

## Abstract

We present a genome assembly from an individual male *Aglais io *(also known as *Inachis io* and *Nymphalis io*) (the European peacock; Arthropoda; Insecta; Lepidoptera; Nymphalidae). The genome sequence is 384 megabases in span. The majority (99.91%) of the assembly is scaffolded into 31 chromosomal pseudomolecules, with the Z sex chromosome assembled. Gene annotation of this assembly on Ensembl has identified 11,420 protein coding genes.

## Species taxonomy

Eukaryota; Metazoa; Ecdysozoa; Arthropoda; Hexapoda; Insecta; Pterygota; Neoptera; Endopterygota; Lepidoptera; Glossata; Ditrysia; Papilionoidea; Nymphalidae; Nymphalinae; Nymphalis; Aglais; *Aglais io* (Linnaeus, 1758) (also known as *Inachis io* and *Nymphalis io*) (NCBI:txid171585).

## Introduction

The European peacock ( *Aglais io*, synonyms include *Inachis io* and *Nymphalis io*) is a palearctic butterfly species. *A. io* is easily recognised by the large and colourful eyespots on its wings, which act as a defence against avian predators ( Blest, 1957; Vallin *et al*., 2005). It is distributed from temperate Europe to Japan, with larvae feeding on nettles and hops ( *Urtica dioica*, *Urtica urens,* and *Humulus lupulus*). It has recently (end of the 20th century) been introduced to Canada. It overwinters as an adult and it is generally considered as univoltine in the British Isles, although in the south it may display a partial second generation. In southern Europe it has two generations per year, and occasionally a partial third one. It is found throughout the British Isles, although rare in the Outer Hebrides, and has increased in both abundance and occurrence over the last 50 years ( Fox *et al*., 2015). This species is listed as Least Concern in the IUCN Red List (Europe) ( van Swaay *et al*., 2010). *A. io* has 31 pairs of chromosomes ( Maeki & Makino, 1953; Maeki, 1953) and the female is heterogametic (WZ). Male genome size has been estimated at approximately 364Mb using flow cytometry ( Mackintosh *et al*., 2019).

## Genome sequence report

**Figure 1.**
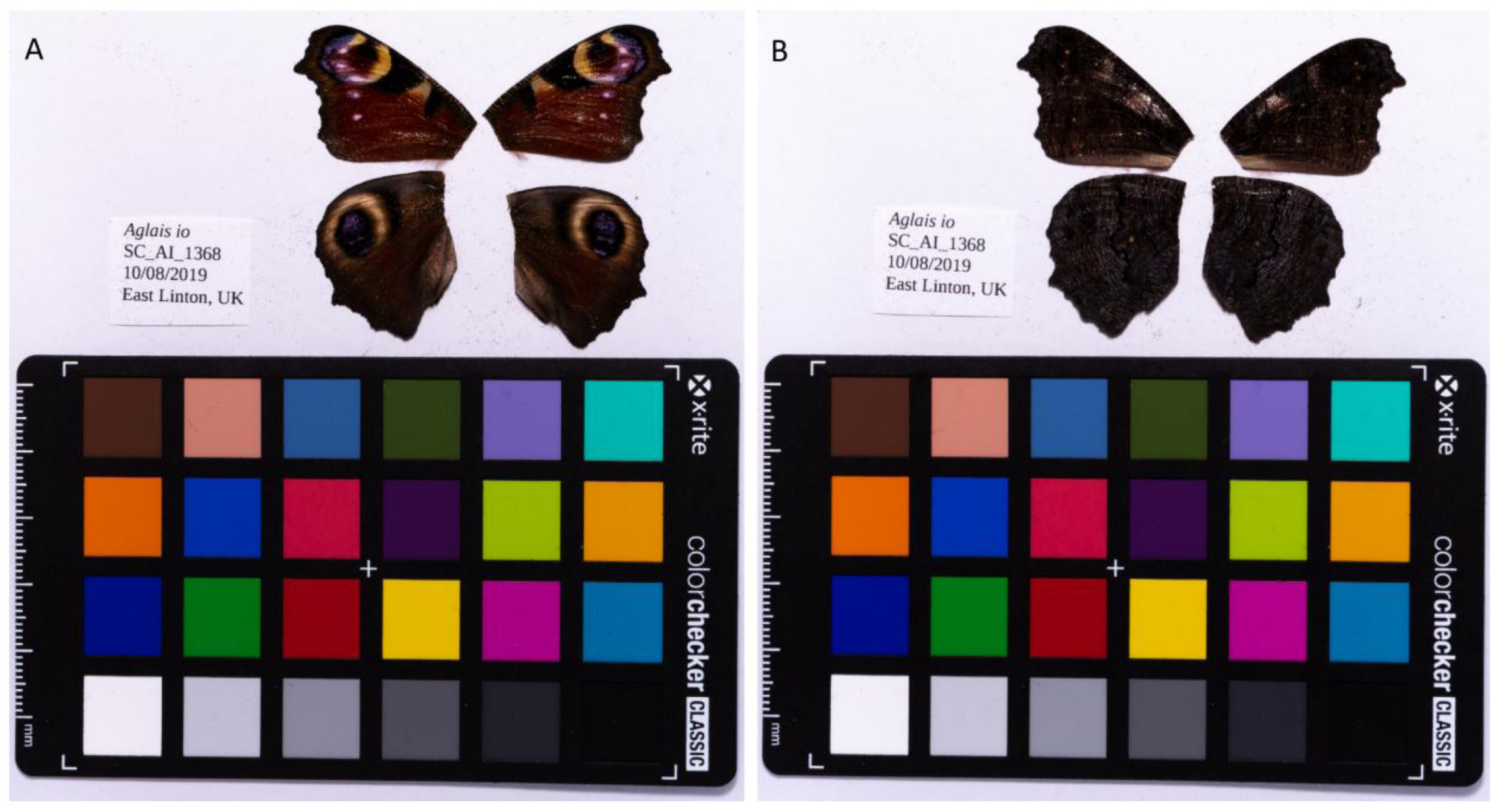
Fore and hind wings of *Aglais io* specimen from which the genome was sequenced. ( **A**) Dorsal surface view of wings from specimen SC_AI_1368 (ilAglIoxx1) from East Linton, used to generate Pacific Biosciences and 10X genomics data. ( **B**) Ventral surface view of wings from specimen SC_AI_1368 (ilAglIoxx1) from East Linton, used to generate Pacific Biosciences and 10X genomics data.

The genome was sequenced from a single male *A. io* ( Figure 1) collected from East Linton, East Lothian, Scotland, UK (latitude 55.977161, longitude -2.667545). A total of 64-fold coverage in Pacific Biosciences single-molecule long reads and 77-fold coverage in 10X Genomics read clouds were generated. Primary assembly contigs were scaffolded with chromosome conformation Hi-C data. Manual assembly curation corrected 13 missing/misjoins and removed three haplotypic duplications, reducing the assembly length by 0.02% and the scaffold number by 16.33%, and increasing the scaffold N50 by 3.06%.

**Figure 2.**
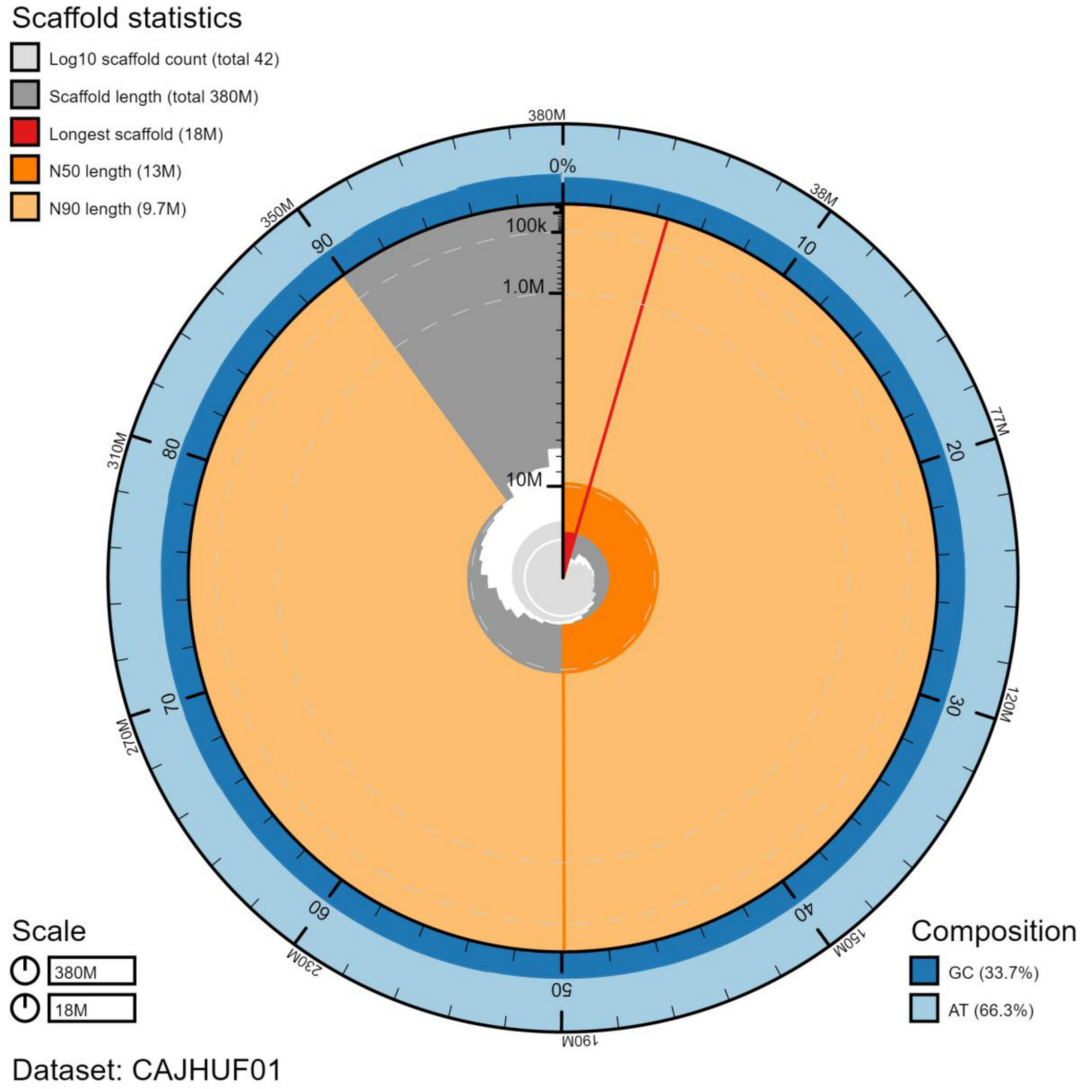
Genome assembly of *Aglais io*, ilAglIoxx1.1: metrics. The BlobToolKit Snailplot shows N50 metrics and BUSCO gene completeness. An interactive version of this figure is available at https://blobtoolkit.genomehubs.org/view/ilAglIoxx1.1/dataset/CAJHUF01/snail.

The final assembly has a total length of 384 Mb in 42 sequence scaffolds with a scaffold N50 of 13 Mb ( Table 1). The majority, 99.91%, of the assembly sequence was assigned to 31 chromosomal-level scaffolds, representing 30 autosomes (numbered by sequence length), and the Z sex chromosome ( Figure 2– Figure 5; Table 2). The assembly has a BUSCO ( Simão *et al*., 2015) v5.1.2 completeness of 98.8% using the lepidoptera_odb10 reference set. While not fully phased, the assembly deposited is of one haplotype. Contigs corresponding to the second haplotype have also been deposited.

**Table 1.** Genome data for *Aglais io*, ilAglIoxx1.1.

*Project accession data*	
Assembly identifier	ilAglIoxx1.1
Species	*Aglais io* (also known as *Inachis io* and *Nymphalis io*)
Specimen	SC_AI_1368, ilAglIoxx1
NCBI taxonomy ID	NCBI:txid171585
BioProject	PRJEB42130
BioSample ID	SAMEA7523149
Isolate information	Male, whole organism
*Raw data accessions*	
PacificBiosciences SEQUEL II	ERR6565934
10X Genomics Illumina	ERR6002579, ERR6002580, ERR6003036, ERR6003037
Hi-C Illumina	ERR6002578
Illumina PolyA RNA-Seq	ERR6286702
*Genome assembly*	
Assembly accession	GCA_905147045.1
*Accession of alternate haplotype*	GCA_905147125.1
Span (Mb)	384
Number of contigs	52
Contig N50 length (Mb)	13
Number of scaffolds	42
Scaffold N50 length (Mb)	13
Longest scaffold (Mb)	16
BUSCO * genome score	C:98.8%[S:98.5%,D:0.3%],F:0.4%,M:0.9%,n:5286
*Genome annotation*	
Number of protein-coding genes	11,420
Average length of protein-coding gene (bp)	1609
Average number of exons per gene	9
Average exon size (bp)	287
Average intron size (bp)	1946

*BUSCO scores based on the lepidoptera_odb10 BUSCO set using v5.1.2. C= complete [S= single copy, D=duplicated], F=fragmented, M=missing, n=number of orthologues in comparison. A full set of BUSCO scores is available at https://blobtoolkit.genomehubs.org/view/ilAglIoxx1.1/dataset/CAJHUF01/busco.

**Figure 3.**
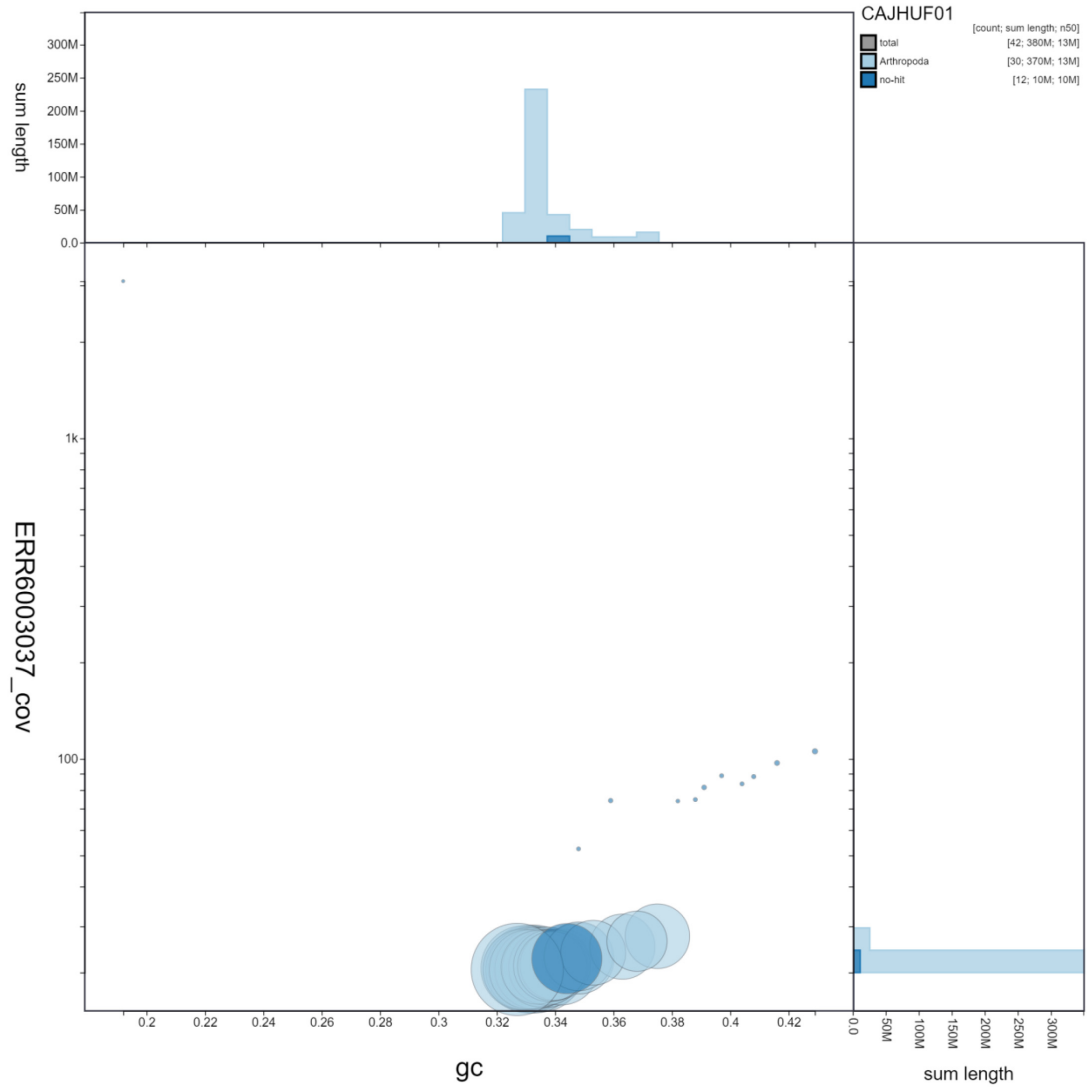
Genome assembly of *Aglais io*, ilAglIoxx1.1: GC coverage. BlobToolKit GC-coverage plot. Chromosomes are coloured by phylum. Circles are sized in proportion to chromosome length. Histograms show the distribution of chromosome length sum along each axis. An interactive version of this figure is available at https://blobtoolkit.genomehubs.org/view/ilAglIoxx1.1/dataset/CAJHUF01/blob.

**Figure 4.**
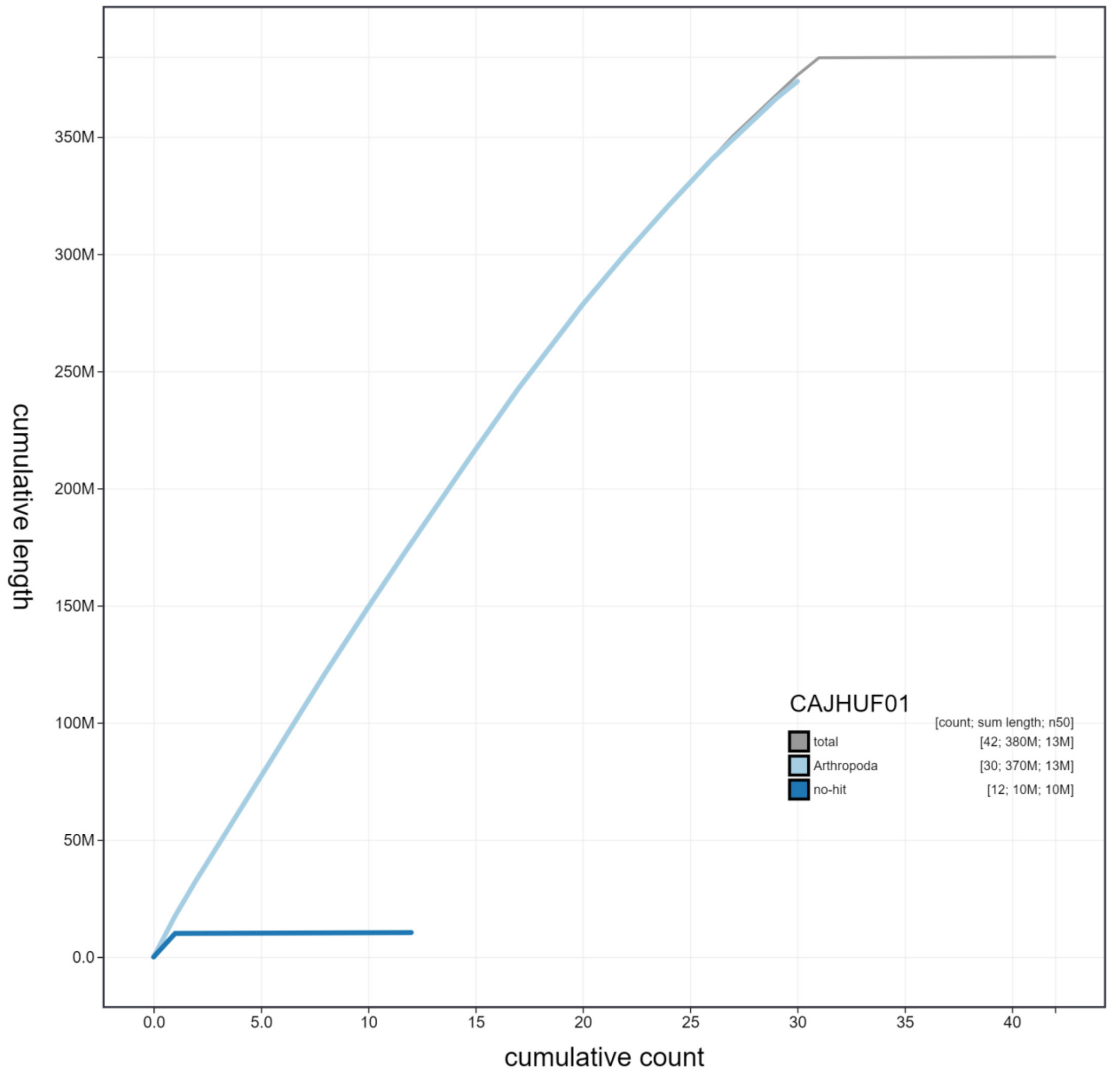
Genome assembly of *Aglais io*, ilAglIoxx1.1: cumulative sequence. BlobToolKit cumulative sequence plot. The grey line shows cumulative length for all chromosomes. Coloured lines show cumulative lengths of chromosomes assigned to each phylum using the buscogenes taxrule. An interactive version of this figure is available at https://blobtoolkit.genomehubs.org/view/ilAglIoxx1.1/dataset/CAJHUF01/cumulative.

**Figure 5.**
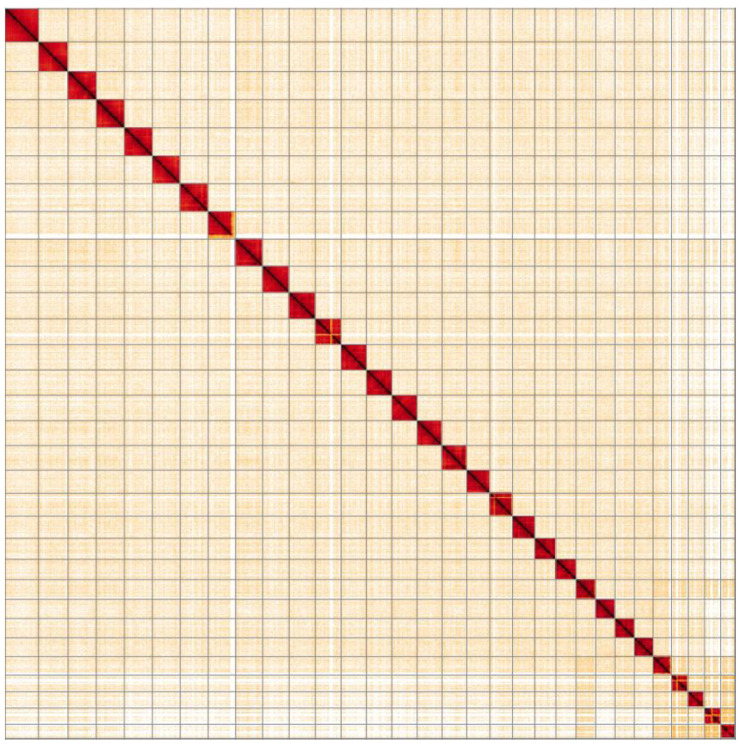
Genome assembly of *Aglais io*, ilAglIoxx1.1: Hi-C contact map. Hi-C contact map of the ilAglIoxx1.1 assembly, visualised in HiGlass.

## Genome annotation

The Ensembl gene annotation system ( Aken *et al*., 2016) was used to generate annotation for the *A. io* assembly ( GCA_905147045.1; Table 1). The annotation was created primarily through alignment of transcriptomic data to the genome, with gap filling via protein to-genome alignments of a select set of proteins from UniProt ( UniProt Consortium, 2019) and OrthoDB ( Kriventseva *et al*., 2008). Prediction tools, CPC2 ( Kang *et al*., 2017) and RNAsamba ( Camargo *et al*., 2020), were used to aid determination of protein coding genes.

## Methods

The male *A. io* specimen SC_AI_1368 was collected from East Linton, East Lothian, Scotland, UK (latitude 55.977161, longitude -2.667545) by Konrad Lohse, University of Edinburgh, using a net. The specimen was snap-frozen in liquid nitrogen.

DNA was extracted at the Wellcome Sanger Institute (WSI) Scientific Operations core from the whole organism using the Qiagen MagAttract HMW DNA kit, according to the manufacturer’s instructions. RNA was extracted in the Tree of Life Laboratory at the WSI using TRIzol (Invitrogen), according to the manufacturer’s instructions. RNA was then eluted in 50 μl RNAse-free water and its concentration RNA assessed using a Nanodrop spectrophotometer and Qubit Fluorometer using the Qubit RNA Broad-Range (BR) Assay kit. Analysis of the integrity of the RNA was done using Agilent RNA 6000 Pico Kit and Eukaryotic Total RNA assay.

Pacific Biosciences HiFi circular consensus and 10X Genomics read cloud DNA sequencing libraries, in addition to PolyA RNA-Seq libraries, were constructed according to the manufacturers’ instructions. DNA and RNA sequencing was performed by the Scientific Operations core at the WSI on Pacific Biosciences SEQUEL II (HiFi), Illumina HiSeq X (10X) and Illumina HiSeq 4000 (RNA-Seq) instruments. Hi-C data were generated using the Qiagen EpiTect Hi-C kit and sequenced on HiSeq X.

Assembly was carried out with HiCanu ( Nurk *et al*., 2020); haplotypic duplication was identified and removed with purge_dups ( Guan *et al*., 2020). One round of polishing was performed by aligning 10X Genomics read data to the assembly with longranger align, calling variants with freebayes ( Garrison & Marth, 2012). The assembly was then scaffolded with Hi-C data ( Rao *et al*., 2014) using SALSA2 ( Ghurye *et al*., 2019). The assembly was checked for contamination and corrected using the gEVAL system ( Chow *et al*., 2016) as described previously ( Howe *et al*., 2021). Manual curation was performed using gEVAL, HiGlass ( Kerpedjiev *et al*., 2018) and Pretext. The mitochondrial genome was assembled using MitoHiFi ( Uliano-Silva *et al*., 2021). The genome was analysed and BUSCO scores generated within the BlobToolKit environment ( Challis *et al*., 2020). Table 3 contains a list of all software tool versions used, where appropriate.

**Table 2.** Chromosomal pseudomolecules in the genome assembly of *Aglais io*, ilAglIoxx1.1.

INSDC accession	Chromosome	Size (Mb)	GC%
LR989896.1	1	15.53	33.1
LR989897.1	2	14.85	33.1
LR989898.1	3	14.76	33.3
LR989899.1	4	14.73	33
LR989900.1	5	14.64	32.9
LR989901.1	6	14.64	33.5
LR989902.1	7	14.56	33.3
LR989903.1	8	14.20	33.3
LR989904.1	9	13.92	33.1
LR989905.1	10	13.78	33.3
LR989906.1	11	13.51	33.3
LR989907.1	12	13.37	33
LR989908.1	13	13.34	32.9
LR989909.1	14	13.32	33.7
LR989910.1	15	13.09	33.3
LR989911.1	16	12.94	33.1
LR989912.1	17	12.25	33.5
LR989913.1	18	11.87	34.2
LR989914.1	19	11.73	33.7
LR989915.1	20	11.12	33.4
LR989916.1	21	10.60	33.8
LR989917.1	22	10.35	34.8
LR989918.1	23	10.17	34
LR989919.1	24	10.08	34.4
LR989920.1	25	9.90	33.9
LR989921.1	26	9.71	34.8
LR989922.1	27	8.71	36.3
LR989923.1	28	8.62	35.3
LR989924.1	29	8.55	37.5
LR989925.1	30	7.43	36.8
LR989895.1	Z	17.55	32.7
LR989926.1	MT	0.02	19.3
-	Unplaced	0.33	39.5

**Table 3.** Software tools used.

Software tool	Version	Source
HiCanu	1.0	Nurk *et al*., 2020
purge_dups	1.2.3	Guan *et al*., 2020
SALSA2	2.2	Ghurye *et al*., 2019
longranger align	2.2.2	https://support.10xgenomics.com/genome-exome/software/pipelines/latest/advanced/other-pipelines
freebayes	1.3.1-17-gaa2ace8	Garrison & Marth, 2012
MitoHiFi	1.0	Uliano-Silva *et al*., 2021
gEVAL	N/A	Chow *et al*., 2016
HiGlass	1.11.6	Kerpedjiev *et al*., 2018
PretextView	0.1.3	https://github.com/wtsi-hpag/PretextView
BlobToolKit	2.6.1	Challis *et al*., 2020

The materials that have contributed to this genome note were supplied by a Tree of Life collaborator. The Wellcome Sanger Institute employs a process whereby due diligence is carried out proportionate to the nature of the materials themselves, and the circumstances under which they have been/are to be collected and provided for use. The purpose of this is to address and mitigate any potential legal and/or ethical implications of receipt and use of the materials as part of the research project, and to ensure that in doing so we align with best practice wherever possible.

The overarching areas of consideration are:

- Ethical review of provenance and sourcing of the material;
- Legality of collection, transfer and use (national and international).

Each transfer of samples is undertaken according to a Research Collaboration Agreement or Material Transfer Agreement entered into by the Tree of Life collaborator, Genome Research Limited (operating as the Wellcome Sanger Institute) and in some circumstances other Tree of Life collaborators.

## Data availability

European Nucleotide Archive: Inachis io (European peacock). Accession number PRJEB42130; https://identifiers.org/ena.embl:PRJEB42130.

The genome sequence is released openly for reuse. The *A. io* genome sequencing initiative is part of the Darwin Tree of Life (DToL) project. All raw sequence data and the assembly have been deposited in INSDC databases. Raw data and assembly accession identifiers are reported in Table 1.
