# Supplementary material for: The genome sequence of the European peacock butterfly, Aglais io (Linnaeus, 1758)
Source: Wellcome Open Res. 2021 Oct 12;6:258. [Version 1] doi: 10.12688/wellcomeopenres.17204.1 (PMC9372638; doi:10.12688/wellcomeopenres.17204.1)
Supplement: Peer review history

# Peer review: Reviewer response for version 1

**DOI:** 10.21956/wellcomeopenres.19009.r51901

**Published:** 2022-08-22

**Authors:** Jing Zhang

**Affiliations:** 1 Eugene McDermott Center for Human Growth and Development, University of Texas Southwestern Medical Center, Dallas, TX, USA

The article is well written and clearly describes how authors constructed the high-quality reference genomes of the peacock butterfly with various sequencing techniques. The genome was assembled into chromosomes and annotated for sharing among the scientific community. The only minor question I have is why not sequence the female which includes the W chromosome. And I also highly recommend sequencing female instead of male in future Darwin Tree of Life projects for Lepidoptera if possible. Sequencing females may lead to high-quality assembly of W chromosome, the important sex chromosome which is rarely included in the current Lepidoptera genome assemblies due to high repetitive content. However, the capacity of integration of multiple sequencing techniques by Darwin Tree of Life project has high chance to assemble W chromosome and make a big difference on sex chromosome research in Lepidoptera.

Are sufficient details of methods and materials provided to allow replication by others?

Yes

Is the rationale for creating the dataset(s) clearly described?

Yes

Are the datasets clearly presented in a useable and accessible format?

Yes

Are the protocols appropriate and is the work technically sound?

Yes

Reviewer Expertise:

Genomics, computational biology

I confirm that I have read this submission and believe that I have an appropriate level of expertise to confirm that it is of an acceptable scientific standard.

# Peer review: Reviewer response for version 1

**DOI:** 10.21956/wellcomeopenres.19009.r51820

**Published:** 2022-08-11

**Authors:** Erin O. Campbell

**Affiliations:** 1 Canadian Food Inspection Agency, Ottawa, Canada

This note presents a near-chromosome level genome assembly for the butterfly *Aglais io *using long-read, read cloud, and chromosome conformation sequencing techniques, as well as annotation using RNAseq data. The data production and assembly steps were performed according to current best-practices, and the authors have done their due diligence in the reporting of data processing and assembly statistics, making this a valuable resource for genomic studies of *A. io* and other related species.

Are sufficient details of methods and materials provided to allow replication by others?

Yes

Is the rationale for creating the dataset(s) clearly described?

Yes

Are the datasets clearly presented in a useable and accessible format?

Yes

Are the protocols appropriate and is the work technically sound?

Yes

Reviewer Expertise:

Insect molecular systematics and evolution

I confirm that I have read this submission and believe that I have an appropriate level of expertise to confirm that it is of an acceptable scientific standard.

# Peer review: Reviewer response for version 1

**DOI:** 10.21956/wellcomeopenres.19009.r49816

**Published:** 2022-05-04

**Authors:** Lars Podsiadlowski

**Affiliations:** 1 Centre for Molecular Biodiversity Research, Zoological Research Museum Alexander Koenig, Bonn, Germany

The authors present the genome sequence assembly of a butterfly, *Aglais io*. This work was done in the Darwin tree of life framework. Coverage of sequencing and additional Hi-C data ensure a streamlined assembly procedure. Analysis methods are well chosen and provide maximum quality of the process. The genome metrics and methods are clearly described. This data report can be indexed as it is.

 Maybe the section "Genome annotation" can be part of the methods, or is the provided order of sections a rule of the journal?

Are sufficient details of methods and materials provided to allow replication by others?

Yes

Is the rationale for creating the dataset(s) clearly described?

Yes

Are the datasets clearly presented in a useable and accessible format?

Yes

Are the protocols appropriate and is the work technically sound?

Yes

Reviewer Expertise:

Comparative genomics of insects and fish

I confirm that I have read this submission and believe that I have an appropriate level of expertise to confirm that it is of an acceptable scientific standard.
